# Effect of the Food Matrix on the Survival to the Gastrointestinal Transit of *Lacticaseibacillus rhamnosus* CRL1505: A Randomized, Controlled, Crossover Study

**DOI:** 10.1002/mnfr.70272

**Published:** 2025-09-20

**Authors:** Nicola Mangieri, Viola Termine, Giorgio Gargari, Nicolò Fornasari, Michele Isotti, Susana Salva, Julio Villena, María Pía Taranto, Valentina Taverniti, Susana Álvarez, Graciela Font, Ylenia Zanchetta, Stefania Arioli, Diego Mora

**Affiliations:** ^1^ Department of Food, Environmental and Nutritional Sciences University of Milan Milan Italy; ^2^ Sacco Srl Cadorago Como Italy; ^3^ Centro de Referencia para Lactobacilos (CERELA‐CONICET) San Miguel de Tucumán Argentina

## Abstract

Probiotic survival is crucial for their beneficial effect, but only a few studies have assessed the impact of delivery format on probiotic viability after passage through the gastrointestinal tract.Our study aimed to investigate the survival of *Lacticaseibacillus rhamnosus* (*L. rhamnosus*) CRL1505 through the gastrointestinal transit in healthy volunteers after a daily consumption of at least 1 billion CFUs per day administered as freeze‐dried cells or incorporated into oat‐ and milk‐fermented drinks. The probiotic's survival was evaluated by combining culture‐based and molecular techniques. Our findings revealed that *L. rhamnosus* CRL1505 survival was significantly higher when the probiotic was administered as an oat‐ and as a milk‐fermented drink. The study highlighted the persistence of CRL1505 in a subset of volunteers 1 week after the last administration. The metataxonomic analysis of fecal samples did not reveal significant changes in microbiota diversity among treatment groups, as expected after probiotic administration in healthy subjects.The survival of CRL1505 through the human gastrointestinal tract was demonstrated with and without the protection of food matrices, highlighting the strain's remarkable ability to withstand harsh conditions. Our findings emphasize the importance of food matrix selection for improving probiotic viability and its potential efficacy in functional foods.

## Graphical abstract

We evaluated the impact of food matrix on *Lacticaseibacillus rhamnosus* CRL1505 survival and total quantification, carried out with strain‐specific approaches. Our results confirmed (i) the remarkable ability of CRL1505 to withstand harsh gastrointestinal conditions and (ii) the protective effect of the food matrix.

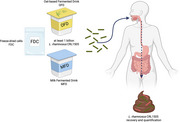

## 1 Introduction

Probiotics, defined as live microorganisms that confer health benefits when consumed in adequate amounts, have gained increasing attention due to their potential to improve gut health, enhance immune function, and prevent various diseases [1, 2]. The effectiveness of probiotics largely depends on their viability and ability to survive storage conditions, processing, and passage through the gastrointestinal tract [3]. Probiotics can be consumed through dairy and nondairy fermented foods and beverages, or as food supplements containing freeze‐dried cells (FDC) [4]. Incorporation into different food matrices, including dairy‐based fermented beverages and/or plant‐based alternatives, might protect the functionality of probiotics and improve their ability to survive gastrointestinal transit (GIT) [4]. In this context, food could act as a buffer protecting probiotics during the GIT, enhancing their survival, viability, and acid and bile tolerance [5, 6]. Dairy‐based fermented beverages (e.g., yogurt, kefir, and probiotic milk drinks) have traditionally been the most common carriers of probiotics. Milk provides an excellent medium for probiotic growth due to its nutrient composition (including proteins, fats, and lactose), which supports bacterial growth and viability [7, 8, 9]. However, the increasing prevalence of lactose intolerance, milk allergies, and changing consumer preferences for plant‐based diets has led to a rise in demand for nondairy probiotic alternatives [10, 11]. Moreover, the growing demand for healthy food is reshaping market trends, driving the development of products that cater to dietary restrictions, allergies, and intolerances while also providing cultural and social comfort [12]. In this context, plant‐based foods offer a sustainable alternative for those people seeking substitutes for animal products or following vegetarian and vegan lifestyles. Common probiotic‐fermented cereals such as maize, sorghum, wheat, oat, millet, barley, and rye are widely used in the production of beverages, gruels, and porridges [13]. Among these cereal options, oat‐based fermented beverages have emerged as a promising alternative due to their nutritional benefits and prebiotic potential. Oats contain β‐glucan, a soluble fiber known to promote gut health and enhance probiotic survival by serving as a substrate for beneficial bacteria [14, 15].

Each of these probiotic delivery methods—fermented dairy and plant‐based beverages, as well as lyophilized formulations—presents unique advantages and challenges. Understanding the impact of these matrices on probiotic viability and health benefits is essential for optimizing their efficacy and expanding their application in functional foods.

*Lacticaseibacillus rhamnosus* (*L. rhamnosus*) CRL1505 [16, 17] is a well‐studied probiotic isolated from goat milk. It has been extensively investigated for its capacity to enhance respiratory immunity in mice, reducing susceptibility to viral and gram‐positive bacterial infections [18, 19, 20, 21], and to decrease the incidence and severity of respiratory infections in children who were administered the probiotic strain [22].

This study aimed to investigate the influence of different food matrices as probiotic vehicles on the survival of the CRL1505 strain after GIT. Thus, we carried out a crossover study in healthy adults, administering *L. rhamnosus* CRL1505 for seven days as FDC or included pre‐fermentation in oat‐ or milk‐based fermented drinks. We quantified viable and cultivable and total CRL1505 strain cells in feces, integrating culture techniques with molecular methodologies employing strain‐specific primers.

## 2 Materials and Methods

### 2.1 Human Intervention Study and Ethical Statement

**FIGURE 1:**
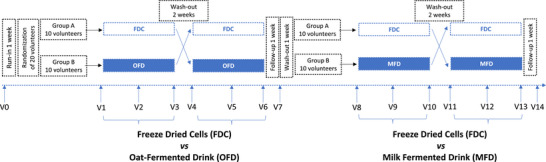
Design of the *survivor* study. Arrows indicate the days of fecal sample collection (when defecation occurred) from volunteers. V, visit for stool delivery to the laboratory for viable recovery analysis.

The influence of the delivery form on the survival of the probiotic strain *L. rhamnosus* CRL1505 during GIT transit was investigated through a series of randomized, controlled crossover studies (Figure 1). The CRL1505 strain belongs to the culture collection of the Reference Center for Lactobacilli (CERELA‐CONICET). The intervention study was carried out from March until June 2023 at the University of Milan (Milan, Italy). The study protocol was approved by the Research Ethics Committee of the Università degli Studi di Milano (opinion 13/23, February 20, 2023). Study title: Effect of the food matrix on the survival of the probiotic strain *L. rhamnosus* CRL1505 in healthy adult subjects (*survivor*). Written informed consent was obtained from all the subjects before recruitment.

Inclusion criteria during recruitment included volunteers of both sexes aged between 18 and 60 who signed informed consent documents. On the other hand, exclusion criteria encompassed gastrointestinal abnormalities (e.g., IBD such as Crohn's disease, and ulcerative colitis), pregnancy, metabolic disorders, primary or secondary immunodeficiency, recent antibiotic usage within 1 month before the screening visit, hypersensitivity, or allergies to any components of the study products, as well as the consumption of probiotic formulations containing *L. rhamnosus* within 1 month prior to the start of the study.

The intervention study consisted of two consecutive crossover studies (Figure 1), each interspaced by 2 weeks of washout. Each treatment foresaw the daily consumption for 7 days of at least 1 billion viable CRL15205 cells as FDC or included in an oat‐fermented drink (OFD) or a milk‐fermented drink (MFD). In the first part of the treatment, we compared FDC and OFD; in the second part, FDC and MFD. A total of 14 fecal samples were collected for each volunteer throughout the studies, according to the scheme reported in Figure 1.

A manual power analysis was conducted based on the formula for a paired *t*‐test. Assuming a two‐tailed significance level of 0.05, a power of 80%, a standard deviation of the difference of 1 log_10_ CFUg^−1^, and an expected effect size of 1 log_10_ CFUg^−1^, a minimum of 8 participants would be required per crossover arm. Our study included more than 8 participants (*n* = 10 per crossover arm), confirming that the sample size was more than adequate to detect biologically relevant differences.

Each volunteer was asked to collect at least 2 g of fecal samples in 100 mL sterile containers (ThermoFisher Scientific, USA), compatible with their bowel habits, before the beginning of each treatment (V1, V4, V8, and V11), after 3–4 consumptions of each probiotic product (V2, V5, V9, and V12), and at the end of the week of each treatment (V3, V6, V10, and V13) (Figure 1). Additionally, two follow‐up samples were collected, 1 week after the first phase and the other after the second study phase (V7, V14) (Figure 1). Throughout the study period, the volunteers were asked to evaluate the frequency and consistency of the stools according to a validated fecal scoring system (Bristol stool scale). According to the instructions provided to the volunteers, all fecal samples were maintained at 4°C until delivery to the laboratory within 24 h, where they were immediately processed for viable recovery.

Probiotic products were consumed under fasting conditions, in the morning at least half an hour before breakfast, or, alternatively, in the evening at least 2 h after the last meal of the day.

### 2.2 Microbial Load of FDC, OFD, and MFD

Three different delivery formats were tested to understand their impact on the survival of *L. rhamnosus* CRL1505 through the GI passage: (1) FDC, (2) oat‐based fermented drink (OFD), or (3) MFD. The OFD provided to the volunteers was produced as a co‐fermentation with the CRL1505 strain and *Streptococcus thermophilus* (*S. thermophilus*) St1, while the MFD also included *Lactobacillus delbrueckii* (*L. delbrueckii*) subsp. *bulgaricus* Ldb1 as a starter culture. All test products were prepared by Sacco Srl (Cadorago, Italy). The OFD products contained as ingredients oat syrup, pea proteins, sunflower seed oil, water, and sea salt (nutritional composition: carbohydrates = 55.5%, fiber = 5.3%, sugars = 36%, fats = 3.7%), whereas the MFD was made with skimmed cow milk and the mentioned starter cultures.

Separate batches of OFD and MFD were prepared for each week of treatment (first and second), while the FDC was delivered as a single batch. The viable cell counts of the CRL1505 strain in 50 mL of the fermented products, that's FDC, OFD, and MFD, are reported in Table 1; each batch of OFD and MFD was counted on the first and last day of administration (Days 1 and 7). The viable cell counts of *S. thermophilus* St1 and *L. delbrueckii* subsp. *bulgaricus* Ldb1 in OFD and MFD are reported in Table S1. To quantify the number of viable cells, 1 mL of OFD and MFD was serially diluted in MRD (Scharlab, Spain) and plated on HHD (Biolife, Italy) agar (15 g L^−1^) for the selective enumeration of homofermentative and heterofermentative lactobacilli and on M17 (BD Difco, USA) agar (15 g L^−1^) supplemented with 2% (w/v) lactose for the enumeration of *S. thermophilus*. The FDC was serially diluted and plated on MRS 10v. To evaluate the stability of the fermented products, the pH was measured at the beginning and at the end of the week of consumption using an XS pH Meter (Carpi, Italy).

**TABLE 1:** Microbial plate count of *L. rhamnosus* CRL1505 in 50 mL OFD and MFD on Day 1 and Day 7 of consumption.

*L. rhamnosus* CRL1505						
Food matrix	Batch	log_10_ CFU/50 mL (Day 1)	log_10_ CFU/50 mL (Day 7)	Average log_10_ CFU/50 mL	pH (Day 1)	pH (Day 7)
OFD	1°	9.52 ± 0.01	9.34 ± 0.06	9.43 ± 0.11	4.15	4.01
	2°	9.11 ± 0.09	9.25 ± 0.05	9.18 ± 0.10	4.14	4.06
**OFD**	**Average**	**9.36 ± 0.23**	**9.30 ± 0.07**	**9.32 ± 0.18^a^**	**4.15**	**4.04**
MFD	1°	9.61 ± 0.18	9.72 ± 0.02	9.66 ± 0.13	4.33	4.20
	2°	9.55 ± 0.06	9.67 ± 0.02	9.61 ±0.08	4.32	4.23
**MFD**	**Average**	**9.59 ± 0.13**	**9.69 ± 0.03**	**9.64 ± 0.04^b^**	**4.33**	**4.22**
**Food matrix**		**log_10_ CFU g^‐1^**				
**FDC**	**Single batch**	**9.33 ± 0.02^a^**				

*Note*: For this analysis, different bottles from each batch were randomly selected. Data are reported as the mean of three biological replicates ± standard deviation. No significant differences were observed in CFU counts between Day 1 and Day 7 of consumption for OFD (paired *t*‐test, *p* value = 0.79) and MFD (paired *t*‐test, *p* value = 0.16). The total viable CRL1505 count was significantly higher in MFD than OFD and FDC (unpaired *t*‐test, *p* value < 0.001).

Abbreviations: FDC, freeze‐dried cells; MFD, milk‐fermented drink; OFD, oat‐fermented drink.

^a,b^Indicate significant differences.

### 2.3 Bacterial Strains, DNA Extraction, and Strain‐Specific Primer Development

Based on the draft genome of *L. rhamnosus* CRL1505 [17], a gene sequence encoding a topoisomerase was identified as a target for strain‐specific primers. qPCR primers were designed using SnapGene (GSL Biotech LLC) and NCBI Primer‐BLAST tools. A preliminary analysis of the strain‐specific primer sequences revealed no matches with closely related *Lactobacillus* spp. or other lactic acid bacteria species. The strain‐specific primer sequences were LRH‐24aTOP‐F (5’–TTACCGGTCAACGAGAAGCC–3’), LRH‐24aTOP‐R (5’–CGCTTCAAATGTGTCGCCAT–3’). The size of the expected amplicon was 263 bp. Amplification was carried out with the following thermal conditions: an initial denaturation at 95°C for 3 min followed by 34 amplification cycles at 95°C for 30 s, 62.2°C for 30 s, and 72°C for 10 s. The optimization of the annealing temperature for strain primers was conducted on DNA of a pure culture of *L. rhamnosus* CRL1505, extracted as reported above.

Primer specificity was tested on 24 *L. rhamnosus* strains and other closely related lactobacilli as listed above (2 strains of *Lactiplantibacillus plantarum* Lp1, a strain of *Lactobacillus gasseri* Lg1, and *Lactobacillus crispatus* Lc1) supplied by Sacco Srl. To obtain pure cultures, a single colony of each strain was cultured in MRS broth (Difco) at 37°C for 24 h in static conditions. Then, each cell suspension was subjected to DNA extraction using the DNeasy UltraClean Microbial DNA Isolation kit (Qiagen, Germany) following manufacturer instructions. DNA extracted was quantified at 260 nm wavelength in a microplate reader with Gen5 Software (BioTek Instruments, USA).

### 2.4 Identification of an Agar Medium for *L. rhamnosus* CRL1505 Recovery

To optimize the recovery of CRL1505 strain viable cells and to avoid the natural bacterial background of fecal samples (namely, culturable cells of other species able to grow on the same medium set for the CRL1505 strain), a series of media and incubation conditions were tested. The protocol was based on suggestions by the Italian Higher Institute of Health for the enumeration of heterofermentative lactobacilli, which foresee the supplementation of vancomycin to the growth medium and its incubation at 37°C–43°C [23]; we tested the growth of the CRL1505 strain in MRS (BD, Difco) agar (15 g L^−1^) supplemented with 0, 1, or 10 µg mL^−1^ vancomycin (Merck) (MRS 0v, MRS 1v, and MRS 10v, respectively) and incubated at 37°C or 43°C for 72 h [23]. A pure culture of CRL1505 or fecal samples, with and without spiking of the probiotic strain, was spread‐plated on the test agar, and plates were incubated at 37°C or 43°C in anaerobic conditions. None of the media and temperature of incubation negatively affected the recovery of CRL 1505 (Table S2), and MRS 10v incubated at 43°C for 72 h showed the least overgrowth of other intestinal species (data not shown). Therefore, MRS 10v was selected for the cultivation of *L. rhamnosus* CRL1505 from fecal samples. According to the spread plating method, the limit of detection of cultivable CRL1505 strain cells was at least 2 log_10_ eCFU g^−1^ of fecal sample.

### 2.5 Processing of Fecal Samples

All collected fecal samples were analyzed to determine the survival of the CRL1505 strain through GI passage.

Volunteers were instructed to keep the procured fecal samples stored at 4°C and deliver them to the laboratory within 24 h of collection. Samples were immediately processed to assess the viable recovery of the probiotic strain, and an aliquot was stored at −80°C until DNA extraction.

For this purpose, 1 g of fecal sample was diluted in 9 mL of MRD (Scharlab, Spain), homogenized in a sterile Stomacher bag for 3 min by means of a Colworth Stomacher 400 Instrument (Seward, UK). Afterward, serial dilutions of the sample were prepared, and at least 3 consecutive dilutions were plated on the optimized MRS agar (MRS 10v) and incubated at 43°C for 72 h. After incubation, the biomass from each plated dilution was independently collected with a sterile loop and resuspended in a solution of the QIAsymphony PowerFecal Pro DNA Kit (Hilden, Germany). These suspensions were stored at −80°C until the DNA extraction was performed using an automated system, QIAsymphony (Qiagen, Germany), in accordance with the manufacturer's instructions.

For the total quantification of the probiotic cells by strain‐specific primer set used in a qPCR assay, all fecal samples obtained from a single subject were thawed and processed at the same time for DNA extraction. After the sample homogenization, 100 mg of feces were weighed and processed with the QIAsymphony PowerFecal Pro DNA Kit (Qiagen, Germany) according to the manufacturer's instructions.

After isolation, DNA was quantified as described above. Then, each DNA was diluted at 20 ng µL^−1^ and stored at −80°C until molecular analysis by qPCR.

### 2.6 Quantification of Viable and Total *L. rhamnosus* CRL1505 by qPCR

qPCR analysis was performed in a final volume of 15 µL containing 7.5 µL of EvaGreen Supermix (Bio‐Rad Laboratories, Italy), 0.5 µM of each primer, and 100 ng of DNA template. The analysis of melting curves was conducted using Bio‐Rad CFX Manager 3.1 software to validate the specificity of the amplification product.

DNA extracted from the colonies was used for the survival assessment of probiotic cells after GIT transit. The DNA from the highest dilution giving a positive signal in qPCR was used to calculate the “estimated minimum CFU number” (eCFU) [24] of the probiotic strain.

For the absolute quantification of the CRL1505 strain in the fecal samples, a standard calibration curve was prepared [25]. Briefly, serial 10‐fold dilutions from a suspension of lyophilized *L. rhamnosus* CRL1505 were prepared in PBS (NaCl 0.15 M, KH_2_PO_4_ 1 mM, Na_2_HPO_4_ 3 mM, pH 7.4) and quantified by flow cytometry. Cell suspension was stained with SYTO 24 0.1 µM and propidium iodide 0.2 µM, according to ISO 19344 – IDF 232 [25], incubated at 37°C for 15 min, and analyzed by Accuri C6 Plus (BD Biosciences, New Jersey, USA). Based on the cell quantification, increasing amounts of *L. rhamnosus* CRL1505 (*n* = 8; 1 to 8 log_10_ FU) were added to 1 g of a fecal sample negative for the presence of *L. rhamnosus* species to create the points of the standard curve. To confirm the absence of *L. rhamnosus* species in the fecal samples used for the standard curve, we used the following primers: *rham* (5’–TGCATCTTGATTTAATTTTG–3’) and Y2 (5’–CCCACTGCTGCCTCCCGTAGGAGT–3’) [26] and the following thermal conditions: an initial denaturation at 95°C for 3 min, followed by 34 cycles at 95°C for 30 s, 54°C for 30 s, and 72°C for 10 s. After the spiking with a known amount of CRL1505 strain cells, each sample was used for DNA extraction as described above. After quantification, DNA was diluted to 20 ng/µL, and 5 µL of each point of the calibration curve was used for qPCR analysis. The experiment was conducted in duplicate in a final volume of 15 µL, consisting of 7.5 µL of EvaGreen Supermix (Bio‐Rad, Hercules, California, USA) and 0.5 µM of each primer. The standard curve was generated by plotting the Cq values of each point, run in duplicate, against the logarithm (log_10_) of the corresponding cell count.

### 2.7 Metataxonomy Analysis

To assess the impact of probiotic administration in different food matrices on the gut microbiota composition of volunteers, we applied a metataxonomic approach through 16S rRNA gene profiling on total DNA extracted from fecal samples of healthy subjects. DNA was extracted from fecal samples collected both before and after the intervention study. The V3‐V4 variable regions of the 16S rRNA gene were sequenced using the Illumina HiSeq 2500 rapid run platform, following a two‐step PCR protocol [27]. The forward primer used was 5'–ACTCCTRCGGGAGGCAGCAG–3', and the reverse primer was 5'–GGACTACHVHHHTWTCTAAT–3'. The resulting 16S rRNA gene sequencing data were processed and analyzed using QIIME2 [28], incorporating the DADA2 algorithm [29] for quality filtering and sequence variant detection, alongside taxonomic assignment using the SILVA 138–99 database. We calculated α‐diversity indices (Chao1, Simpson, inverse Simpson, and Shannon) to evaluate intrasubject diversity and β‐diversity indices to assess intersubject diversity. The metadata associated with this study has been deposited in the European Nucleotide Archive of the European Bioinformatics Institute under accession code PRJEB89811.

### 2.8 Statistical Analysis

The statistical analysis of cultivability was conducted using the Chi‐square test with R statistical software, version 4.3.1. The Bristol stool form scale was analyzed statistically using the Wilcoxon test for paired samples. The absolute quantification obtained through qPCR was assessed using nonparametric repeated measures ANOVA with the R core team's “nparLD” library. Regarding the taxonomic profiling analyses, R statistical software was also employed, as previously mentioned, to compare differences within the same matrix before and after treatment. Additionally, LEfSe [30] was applied to compare pre and posttreatment across all matrices.

## 3 Results

### 3.1 Probiotic Counts in Test Products

To test the efficacy of the matrix on the delivery of the *L. rhamnosus* CRL1505 probiotic strain, an interventional study was carried out by recruiting a total of 22 healthy adults of both genders (10 females, 10 males), ranging in age from 22 to 57 years old, with a mean age of 35 ± 11 (Table S3), randomly assigned to the following treatment: (i) FDC, (ii) oat‐based fermented drink (OFD) (produced with *S. thermophilus* and CRL 1505), (iii) milk‐based fermented drink (MFD) (produced with yogurt starter cultures and CRL 1505 strain). According to the study protocol (Figure 1), in the first part of the study we compared the probiotic delivery by FDC versus OFD, while in the second part by FDC versus MFD. Volunteer compliance, as determined by verbal assessment, was almost 100%. Indeed, 20 out of 22 volunteers completed the study according to the protocol; two volunteers dropped out because of an antibiotic therapy.

Volunteers were asked to consume FDC, OFD, or MFD containing at least 1 billion CFU per dose (1 g FDC, 50 mL OFD, or MFD). Each batch of production of OFD and MFD was evaluated at the start of each period of consumption (V1–V3 and V4–V6 for OFD, V8–V10 and V11–V13 for MFD) (Table 1). Our results indicate that the consumption of 50 mL of OFD or MFD determined on average an ingestion of 9.33 (±0.18) and 9.65 (±0.04) log_10_ CFU/dose, respectively. Similarly, the consumption of 1 g of FDC determined an ingestion of 9.33 (±0.02) log CFU/dose. The analysis of the CRL1505 cultivability at the end of each period of consumption of OFD and MFD confirmed the microbiological and the pH stability of the products used (Table 1). Moreover, these data indicated that when administered through MFD, volunteers assumed daily a slightly higher number of CRL1505 live cells. Finally, the microbiological analysis of the products carried out before and after the administration to the volunteers confirmed the presence of *S. thermophilus* in OFD and MFD*, L. delbrueckii* subsp. *bulgaricus* only in MFD (Table S4), and the absence of pathogens and other undesired microorganisms that could affect the stability and the safety of OFD and MFD (Table S3).

### 3.2 Assessment of Culturable CRL1505 Cells After GIT

For evaluating GIT survival of the CRL1505 probiotic strain, we combined cultivability assessment on MRS 10v agar medium with the strain‐specific qPCR. In this way, we determined the log_10_ eCFU g^−1^ for CRL1505 after GIT.

**FIGURE 2:**
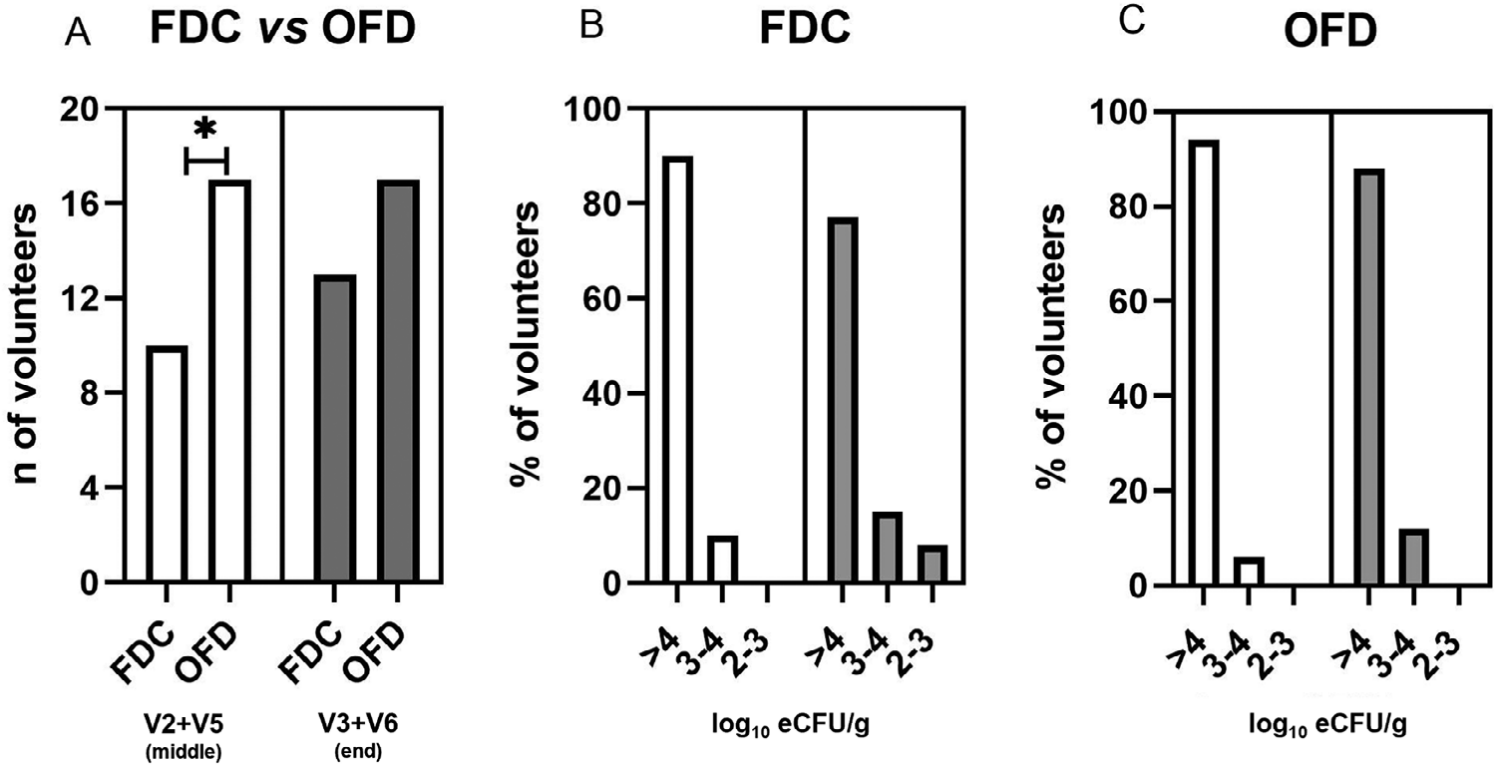
Cultivability of *L. rhamnosus* CRL1505 in fecal sample confronting FDC and OFD (*n* = 18). (A) Number of volunteers positive for the presence of cultivable cells of CRL1505 strain. ^*^Significant differences (*p* < 0.05). (B) Log_10_ eCFU g^−1^ of the CRL1505 strain in fecal samples after FDC consumption. (C) Log_10_ eCFU g^−1^ of the CRL1505 strain in fecal samples after OFD consumption. White and gray bars represent the number of volunteers positive at V2 + V5 (middle, after 3–4 consumptions) or at V3 + V6 (end of the treatment), respectively. Statistical analysis was performed using the Chi‐square test.

In the first phase of the study, we compared FDC versus OFD in a crossover protocol involving 18 volunteers. Indeed, after a 1‐week run‐in at V1, fecal samples from 2 out of 20 volunteers were already positive for the presence of CRL1505. Therefore, samples from these 2 subjects were excluded from the comparison of FDC and OFD. For all other fecal samples, no CRL1505 cells were detected as cultivable cells at V1. After 3–4 days of probiotic administrations (V2), CRL1505 was recovered in 10 (55%) and 17 (94%) out of 18 subjects after consumption of FDC or OFD, respectively. The fecal analysis at the end of the consumption (V3) revealed an increase in positive subjects up to 13 out of 18 (72%) after FDC administration; instead, no variation was observed in the number of volunteers for the presence of cultivable CRL1505 after 1 week of OFD consumption. Statistical analysis revealed significant differences between the two groups only after 3–4 consumptions (V2 and V5) (*p* < 0.05) and not at the end of the treatment (V3 and V6) (Figure 2A). By combining spread plating and strain‐specific qPCR assay, we determined the CRL 1505 log_10_ eCFU g^−1^ (Figure 2B,C). Our analysis revealed that, among CRL1505‐positive subjects, at V2 + V5, 90% of volunteers had at least 4 log_10_ eCFU g^−1^ when consuming CRL 1505 as FDC, with the remaining 10% ranging between 3 and 4 log_10_ eCFU g^−1^. At V3 + V6, 77% of the subjects maintained at least 4 log_10_ eCFU g^−1^, 15% were within the 3–4 log_10_ eCFU g^−1^ range, and 8% fell between 2 and 3 log_10_ eCFU g^−1^. When consuming OFD, after 3–4 consumptions (V2 + V5), 94% of the volunteers had at least 4 log_10_ eCFU g^−1^, and 6% had at least 3–4 log_10_ eCFU g^−1^. At the end of the study (V3 + V6), 88% still had at least 4 log_10_ eCFU g^−1^, and 12% were in the 3–4 log_10_ eCFU g^−1^ range (Figure 2A). It is worth mentioning that, after the washout of 2 weeks and before the crossover, 1 out of 11 subjects remained positive for the presence and cultivability of CRL1505 after 1‐week consumption of FDC; conversely, 2 out of 7 subjects remained positive for the cultivability of the probiotic after the consumption of OFD. Moreover, at the end of the follow‐up lasting 1 week (V7), 3 out of 11 subjects remained positive for the presence of viable and cultivable CRL1505 only after OFD consumption.

**FIGURE 3:**
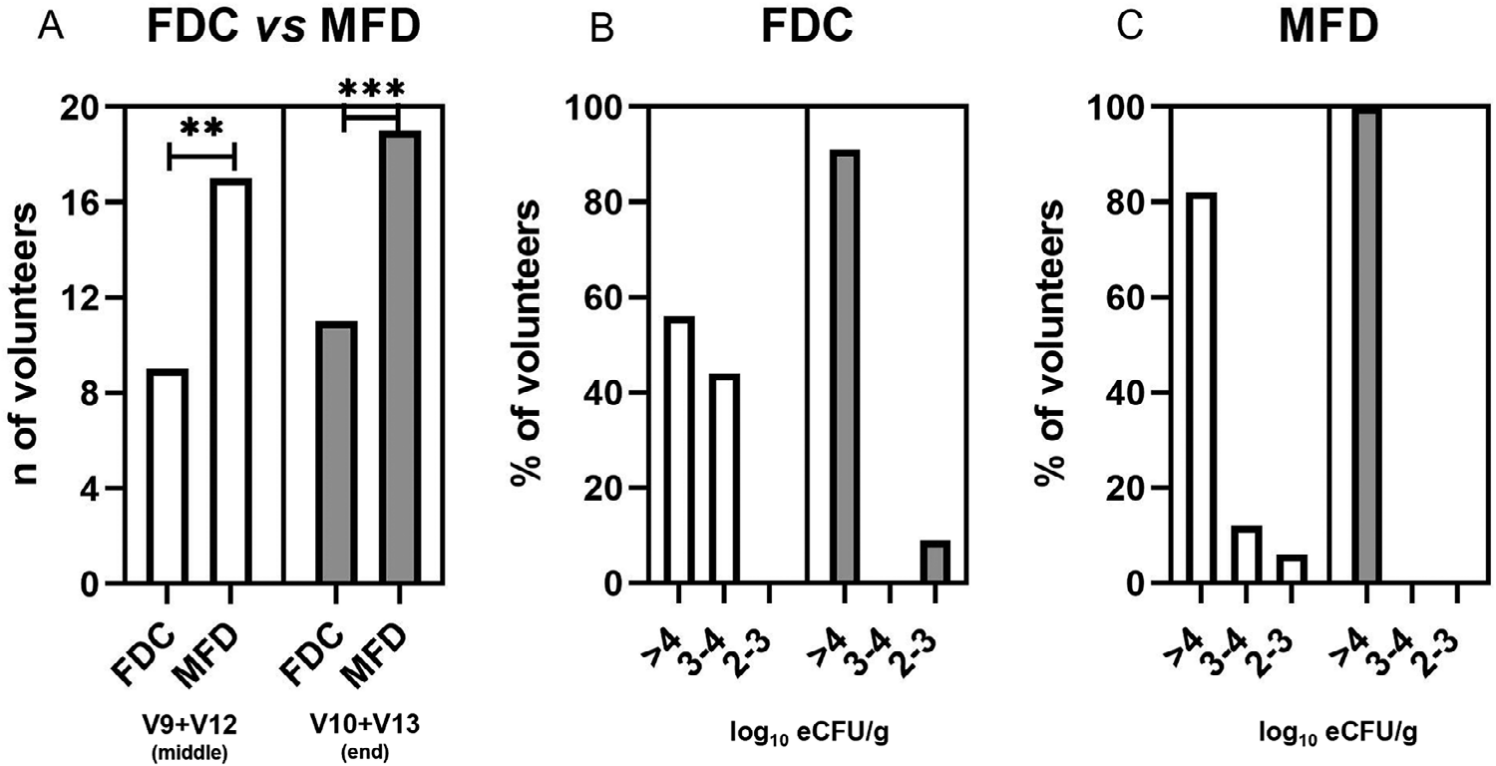
Cultivability of *L. rhamnosus* CRL1505 in fecal sample confronting FDC versus MFD (*n* = 19). (A) Number of volunteers positive for the presence of cultivable cells of CRL1505. ^**^Significant differences (*p* < 0.01); ^***^significant differences (*p* < 0.001). (B) Log_10_ eCFU g^−1^ of CRL1505 strain in fecal samples after FDC consumption. (C) Log_10_ eCFU g^−1^ of the CRL1505 in fecal samples after MFD consumption. White and gray bars represent the number of volunteers positive at V9 + V12 (middle, after 3–4 consumptions) or at V10 + V13 (end of the treatment), respectively. Statistical analysis was performed using the Chi‐square test.

In the second part of the study, we compared FDC versus MFD with a crossover protocol involving 19 out of 20 volunteers (Figure 3). Indeed, at V8, one volunteer remained positive for cultivable probiotic cells (CRL 1505 3–4 log_10_ eCFU g^−1^). Our data showed that FDC consuming group had 9 out of 19 positive subjects (47%) in the middle (V9 and V12) of the treatment and 11 out of 19 (58%) at the end (V10 and V13) (Figure 3A). In contrast, the MFD consuming group achieved a higher number of positive subjects, namely 17 out of 19 (89%) in the middle (V9 and V12) and 19 out of 19 (100%) at the end of the consumption (V10 and V13). Statistically significant differences (*p* < 0.05) between groups (FDC vs. MFD) were observed after 3–4 consumptions and at V10 and V13 (*p* < 0.005) (Figure 3A).

The evaluation of the CRL1505 eCFU g^−1^ after the GIT revealed that 56% of the FDC consuming group had at least 4 log_10_ eCFU/g and 44% at least 3–4 log_10_ eCFU g^−1^ at V9–V12 of the probiotic administration. At the end of the study (V10 and V13), 91% of subjects had at least 4 log_10_ eCFU g^−1^, and 9% had 2–3 log_10_ eCFU g^−1^ (Figure 3B). In volunteers assuming MFD, we found that 82% and 100% of the subjects had at least 4 log_10_ CFU g^−1^ at V9 and V12 and at V10 and V13, respectively (Figure 3C). Analogously to the first crossover, after the washout and before the crossover, 2 out of 11 and 1 out of 8 were positive at V11 after consumption of FDC and MFD, respectively. Finally, at V14, after a 1‐week follow‐up at the end of the treatment (V14), 3 out 11 (from FDC group) and 1 out of 8 (from the MFD group) remained positive (2–3 log_10_ eCFU g^−1^) for CRL1505 cultivability in fecal samples.

### 3.3 Quantification of CRL1505 DNA in Fecal Samples

For the molecular quantification, strain‐specific primers were set up. For the absolute quantification of the probiotic strain in fecal samples, a calibration curve was prepared. The calibration curve showed a linear increase in the range between 4 and 8 log_10_ cells g^−1^, with a correlation coefficient (*R* ^2^) of 0.9958 and primer efficiency of 107%. Also, the limit of quantification was 4 log_10_ cells g^−1^ of feces (Figure S1).

**FIGURE 4:**
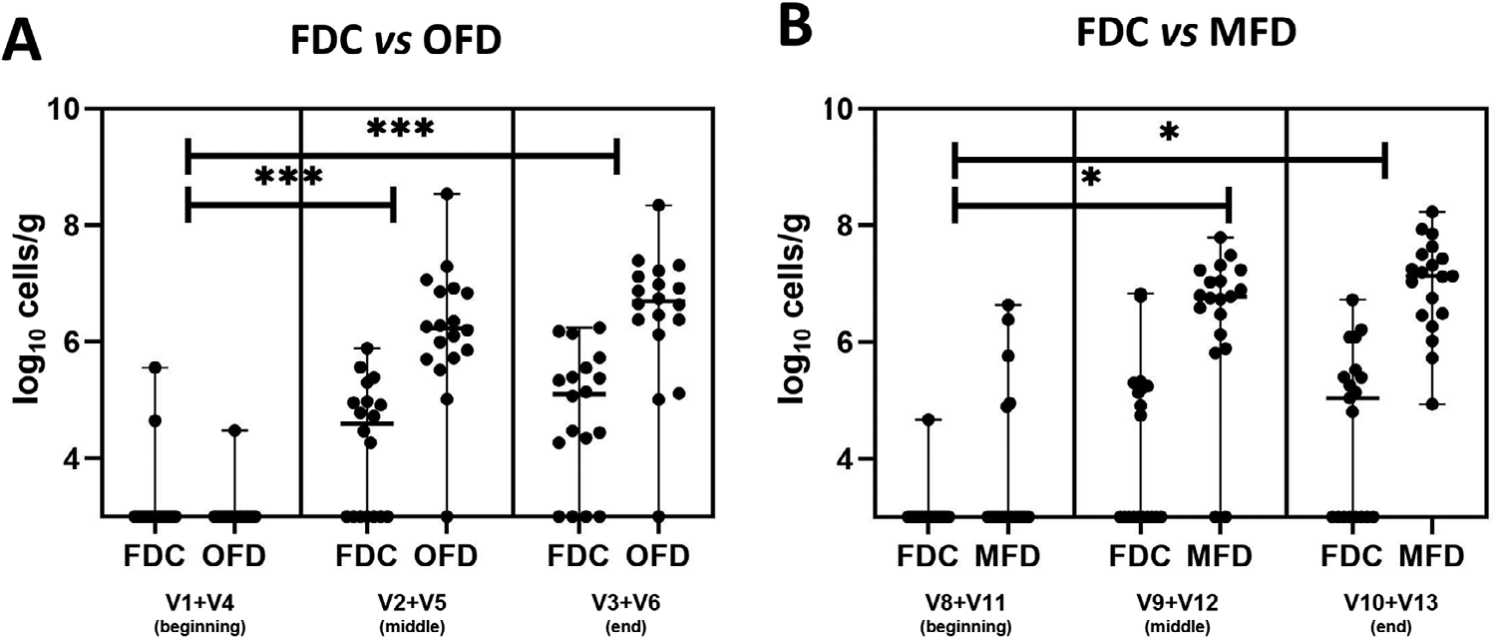
Total CRL1505 cell counts in fecal samples of volunteers of the *survivor* study. (A) FDC versus OFD (*n* = 18). (B) FDC versus MFD (*n* = 19). The statistical analysis was performed using nonparametric ANOVA with repeated measures. Dots represent individual values. The group bars represent the median. ^*^Significant differences (*p* < 0.05); ^***^significant differences (*p* < 0.001).

Fecal samples collected during the study were used for the total quantification of CRL1505 based on strain‐specific qPCR assay. In the first part of the study, confronting FDC versus OFD (Figure 4A), quantification of probiotic cells in fecal samples of a total of 18 volunteers revealed that, at the end of FDC consumption and in the middle of the treatment (V2 and V5), 11 out of 18 were positive for the presence of the probiotic, with a median of 4.78 log_10_ cell g^−1^ feces (wet weight). Moreover, the lowest and highest probiotic cell densities measured at V2 and V5 were 4.3 and 5.9 log_10_ cells g^−1^ fecal sample, respectively. In the OFD consuming group, 17 out of 18 volunteers were positive in the middle of the treatment, with a median of 6.2 log_10_ cells g^−1^ feces, and with the lowest and highest cell density measured between 5.0 and 8.5 log_10_ cells g^−1^ fecal sample, respectively. Analyses on fecal samples at the end of the probiotic consumption (V3 and V6) allowed us to quantify CRL1505 with a median of 5.3 and 6.7 log_10_ cells g^−1^ fecal sample in FDC and OFD groups, respectively. The lowest and highest probiotic cell densities were 4.3 and 6.2 log_10_ cells g^−1^ feces after FDC consumption, whereas they were 5.0 and 8.3 log_10_ cell g^−1^ feces after OFD consumption. Statistical analysis (nonparametric ANOVA with repeated measures) revealed significant differences (*p* < 0.005) when comparing probiotic quantification trends between FDC and OFD from the beginning to the middle of the intervention (V1 and V4 vs. V2 and V5) and from the beginning to the end (V1 and V4 vs. V3 and V6). Conversely, no significant differences were found in the trend from the middle to the end of the treatment (V2 and V5 vs. V3 and V6) (Figure 4A). It is worth mentioning that 1 out of 11 and 2 out of 7 volunteers remained positive for the presence of CRL1505 after 2 weeks of washout (V4) after FDC and OFD consumption, respectively, and after the 1‐week follow‐up, 3 out of 18 volunteers were positive for the CRL1505 strain presence in fecal samples, confirming the results obtained by culturing.

In the second part of the study, we carried out the comparison between FDC and MFD (Figure 4B). At V8, after a 1‐week follow‐up and 1‐week washout, one of the volunteers was positive for the presence of CRL1505 cells, in agreement with viable cell count data. In the middle of the treatment (V9 and V12), 9 out of 19 volunteers were positive for the presence of probiotic cells after FDC administration, with a median of 5.0 log_10_ cells g^−1^ feces, and with the lowest and highest measured values assessed at 4.7 and 6.8 log_10_ cells g^−1^ feces, respectively. Seventeen out of 19 volunteers of the MFD group resulted positive for CRL 1505 cells, with a median of 6.8 log_10_ cell g^−1^ feces, and the lowest and highest values of 5.8 and 7.8 log_10_ cell g^−1^ feces, respectively. Analyses of fecal samples at the end of the probiotic consumption (V10 and V13) showed the presence of CRL 1505 in 8 out 19 volunteers after FDC consumption, with a median of 5.1 log_10_ cells g^−1^ feces, and lowest and highest values of 4.8 and 6.7 log_10_ cells g^−1^ feces, respectively. Conversely, all volunteers were positive for the presence of probiotic cells, with a median of 7.1 log_10_ cell g^−1^ fecal sample, and lowest and highest values of 4.9 and 8.2 log_10_ cells g^−1^ feces, respectively. Statistical analysis (nonparametric ANOVA with repeated measures) revealed significant differences (*p* < 0.005) when comparing probiotic quantification trends between FDC and MFD from the beginning to the middle of the intervention and from the beginning to the end (V8 + V11 vs. V9 + V12, and V8 + V11 vs. V10 + V13). Conversely, no significant differences were found in the probiotic quantification trend from the middle to the end of the treatment (V9 + V12 vs. V10 + V13) (Figure 4B). Noteworthy, 4 out of 11 volunteers remained positive for the presence of CRL1505 after a 1‐week follow‐up (V14), 3 of them after MFD consumption and 1 after FDC administration, as per the cultured data.

### 3.4 Defecation Frequency and Stool Consistency

Volunteers were asked to fill in a questionnaire to evaluate the number of daily defecations and stool consistency. Statistical analysis (Wilcoxon test) carried out on collected data revealed no significant differences for either parameter. Indeed, the daily defecation frequency was not significantly influenced by probiotic consumption through the different matrices. The median value remained constant at 1 for all groups. Similarly, the median values of stool consistency were not impacted by probiotic consumption across the various matrices, with a median value of 3 for the MFD and 4 for the OFD and the FDC at both time points. Further analysis using the Wilcoxon test to compare different consumption times also revealed no significant differences (Tables S4 and S5). Monitoring gastrointestinal symptoms during the weeks of treatment with FDC, OFD, or MFD did not reveal the onset of symptoms that would have induced any of the volunteers to withdraw from the study (data not shown).

### 3.5 Taxonomic Profile of Gut‐Associated Bacteria After *L. rhamnosus* CRL1505 Administration

No significant differences were observed in alpha‐diversity or beta‐diversity (Unifrac) indices between the treatment groups. Moreover, in terms of taxonomic profiling (Table 2), we observed a general increase in *L. rhamnosus* species following treatments with the OFD and MFD, but not with the FDC. Moreover, the OFD consumption was accompanied by a decrease of the Christensenellaceae family. These findings, identified through the Wilcoxon test, were corroborated by LEfSE analyses. Similarly, the FDC intake primarily resulted in an increase in the *Lactobacillus* genus and in a decrease of unknown bacteria belonging to the Lachnospiraceae family, as indicated by both the Wilcoxon test and LEfSE analyses (Table 2).

**TABLE 2:** LDA scores of the profiling analysis carried out for FDC, OFD, and MFD groups pre and posttreatment.

	*p* value	Mean		LDA
**MFD**		V8–V11	V10–V13	
p__Firmicutes; c__*Bacilli*; o__Lactobacillales; f__*Lactobacillaceae*; g__*Lactobacillus*; s__*Lactobacillus rhamnosus*	0.036	0.066	0.964	2.98
**OFD**		V1–V4	V3–V6	
p__Firmicutes; c__*Bacilli*; o__Lactobacillales; f__*Lactobacillaceae*; g__*Lactobacillus*; s__*Lactobacillus_rhamnosus*	0.012	0.066	0.964	2.56
p__Firmicutes; c__Clostridia; o__*Christensenellales*; f__Christensenellaceae.ASV_0848	0.022	0.321	0.042	2.32
**FDC**		V1–V4/V8–V11	V3–V6/V10–V13	
p__Firmicutes; c__*Bacilli*; o__Lactobacillales; f__*Lactobacillaceae*; g__*Lactobacillus*	0.020	0.423	0.833	2.29
p__Firmicutes; c__Clostridia; o__Lachnospirales; f__Lachnospiraceae; g__uncultured; s__uncultured_bacterium.ASV_0692	0.022	0.318	0.063	2.12

Abbreviations: FDC, freeze‐dried cells; LDA, linear discriminant analysis; MFD, milk‐fermented drink; OFD, oat‐fermented drink.

## 4 Discussion

Probiotics can be consumed in a variety of forms, such as different dietary supplement formats (e.g., capsules, sachets, and tablets), included as starters in dairy and nondairy fermented drinks, or added at the end of the fermentation process for obtaining fortified foods [2, 31, 32, 33, 34].

The viability of probiotics after GIT passage is considered by many to be a fundamental prerequisite for them to exert health benefits on the host [2, 35]. Fecal recovery of the probiotic on selective or partially selective and/or differential media after oral intake is the standard method to demonstrate their ability to survive the GIT transit. The survival of probiotics through the GIT transit has been shown for many marketed probiotics, such as *L. paracasei* DG [36], *Bifidobacterium animalis* subsp. *lactis* BB‐12 [31], *L. rhamnosus* GG (formerly *Lactobacillus rhamnosus* GG) [37], *L. paracasei* Shirota [38], and even for multispecies formulations [24]. However, very few studies have investigated the effect of the delivery format on the survival of probiotic cells, either in vitro [34] or in vivo [31, 39]. The ability of CRL1505 cells to survive after exposure to low pH was previously assessed in vitro [40].

In this study, we tested the GIT survival of *L. rhamnosus* CRL1505, a probiotic strain shown to prevent respiratory and intestinal infections when administered in yogurt [22, 41]. Prior studies on the CRL1505 strain have focused on its immunobiotic characteristics [19, 21, 22, 23, 24, 25, 26, 27, 28, 29, 30, 31, 32, 33, 34, 35, 36, 37, 38, 39, 40, 41, 42, 43]. Here, we evaluated the matrix effect on the survival of CRL1505 when administered as FDC or included before the fermentation in oat‐fermented (OFD) and milk‐fermented (MFD) drinks. Adult volunteers consumed at least 1 billion CFUs of *L. rhamnosus* CRL1505 every day, specifically 9.32 ± 0.18 log_10_ CFU per 50 mL for OFD, 9.33 ± 0.02 log_10_ CFU per sachet, and 9.45 ± 0.04 log_10_ CFU per 50 mL for MFD groups. The survival of the strain after GIT passage and the total count in fecal samples were evaluated by culture‐based and molecular methods using strain‐specific enumeration techniques. The viable recovery of probiotic cells from feces is challenging due to the microbiological complexity of the samples, composed of hundreds of different microbial species. In our study, we optimized a protocol combining the cultivability on MRS supplemented with 10 µg mL^−1^ vancomycin and incubation at 43°C for 72 h in anaerobic conditions to partially inhibit the growth of other fecal species without significantly interfering with the growth of the CRL1505 strain. In addition, the DNA extraction from the biomass of each plated dilution and from fecal samples provided by the volunteers, combined with strain‐specific primers used in a qPCR assay, allowed us to calculate the eCFU and the total CRL1505 strain cells, respectively.

The survival of the CRL1505 strain, assumed via OFD, showed significant differences in comparison to FDC observed after 3–4 days, but not at the end of the probiotic consumption. Within the fermented OFD group, the probiotic was detected in 94% of the subjects with at least 4 log_10_ eCFU g^−1^, and 12% had a range of 3–4 log_10_ eCFU g^−1^. Conversely, 55% and 72% of the volunteers tested positive for the presence of CRL1505 strain viable cells in fecal samples at V2 and V3, the middle and end of the treatment, respectively. Similarly to the OFD group, most of the subjects were positive with at least 4 log_10_ eCFU g^−1^. As to what could have favored survival of the strain in the OFD matrix, OFD contains functional components like β‐glucans, antioxidant properties, and free fatty acids. In vitro tests have shown that β‐glucans can foster the growth of the probiotic strains *L. rhamnosus* HN001 and *L. rhamnosus* GG [44, 45]. Also, oat β‐glucans were able to protect fresh cells of two *L. rhamnosus* strains when exposed to low pH [46], indicating that fibers could help in maintaining the viability and stability of the probiotic under these conditions [47]. In a clinical trial, utilized a combination of *L. rhamnosus* probiotic strains and prebiotic β‐glucans in a clinical trial aimed at reducing diabetes. The synergy between prebiotics and probiotics suggests potential for the prebiotic component to uphold bacterial viability within probiotic products and enhance their persistence within the gastrointestinal tract [45]. Furthermore, the OFD can serve as a viable alternative for individuals with lactose intolerance, glucose sensitivity, or casein allergies and provides a valuable source of probiotics for vegans. The expansion of probiotic food offerings, extending beyond fermented dairy products, holds the potential to cater to consumers with distinct dietary preferences and requirements, including strict vegetarians, lactose‐intolerant individuals, those with milk protein allergies, or those harboring negative perceptions toward dairy products [48]. This diversification is poised to address the evolving demands of a broad spectrum of consumers [49]. Among the three formulations used, MFD ensured the recovery of the probiotic in all subjects, with at least 4 log_10_ eCFU g^−1^ in the fecal samples in 91% of the volunteers, like what was observed for the OFD matrix. The capability of the strain to survive the GIT was statistically superior to that of the FDC delivery. This effect could be potentially explained by the slightly higher number of viable cells contained in the MFD product. Moreover, this phenomenon could be explained by the significant levels of organic acids, proteins, lipids, and phosphates contained in the fermented milk, which could act as bioprotective agents under GIT conditions [50], as demonstrated in [51], where bifidobacterial cells mixed with fermented milk broth exhibited improved survival under simulated GIT conditions compared to a culture cell suspension. In general, the survival of probiotic cells was higher in OFD and MFD than in FDC. This could be explained by a sort of low pH adaptation of probiotic cells when included in OFD and MFD and a protective effect of the food matrix. Indeed, unlike MFD and OFD, where the food matrix is recognized to positively impact the survival of probiotic cells, FDC were assumed to have no protection against the harsh gastrointestinal conditions. Despite this, the recovery during and at the end of the probiotic treatment is like what was measured for other strains of *L. rhamnosus* consumed with a comparable daily dose and duration of treatment [52]. Also, the low pH levels of the two drinks (4.1 ± 0.1 and 4.3 ± 0.1 for OFD and MFD, respectively) suggest that the bacterial cells in the fermented drink were exposed to acidic conditions for at least 1 week, allowing them to better adapt to gastric conditions. This phenomenon is known as “cross‐adaptation” [53]. Previous research has also indicated that refrigerated storage conditions in a low pH can enhance the resistance of certain probiotics to simulated gastric conditions, as seen with *L. casei* in fruit juice [49]. *Bifidobacterium animalis* subsp. *lactis* incorporated into various food matrices, including fermented milk and nondairy products, displayed higher resistance when preexposed to adverse conditions [54]. Additionally, *L. rhamnosus* E800 exhibited increased tolerance to gastric acidity when cultivated at pH 5.0 compared to pH 5.8 [55].

Besides the survival of the CRL1505 strain, in our study we evaluated the total amount of probiotic cells in fecal samples in the middle and at the end of their consumption. Our results showed significant differences when comparing OFD and MFD to the FDC. At the conclusion of week consumption, the total cell counts were 6.7 and 7.1 log_10_ cells g^−1^ for the OFD and MFD, respectively. These values were significantly higher compared to FDC, which recorded counts of 5.3 and 5.1 log_10_ cells g^−1^ at the end of the first and second phases of the intervention study. This discrepancy in cell counts underscores the resilience and survival advantage of *L. rhamnosus* CRL1505 within the gastrointestinal tract, offering valuable insights into the potential efficacy of these fermented beverages as probiotic delivery vehicles.

Considering the ability to persist in the gut of the volunteers, we investigated the recovery and quantification of the CRL1505 strain after the run‐in, washout, and follow‐up periods of the study. Analysis of V1 fecal samples allowed us to detect, although only at the molecular level, *L. rhamnosus* CRL1505 in fecal samples of 3 out of 20 volunteers. The presence of the probiotic cells even after the run‐in period could be due to previous consumption of products containing the CRL1505 strain. Indeed, during the run‐in period, volunteers were asked not to consume probiotic‐enriched fermented milk or probiotic‐ and prebiotic‐based food supplements. This could indicate the ability of the strain to persist in fecal samples for a period longer than 1 week. Therefore, these 3 subjects were excluded from the analysis of the first part of the study, where we compared FDC and OFD.

The evaluation of probiotic persistence in fecal samples after 1 week of the end of the treatment (V7 and V14) revealed that 3 out of 18 (17%) (FDC vs. OFD) and 4 out of 19 (21%) (FDC vs. MFD) volunteers remained positive for the presence of CRL 1505. Interestingly, the 3 subjects positive at V7 consumed the CRL1505 strain in OFD, while 3 out of 4 positives at V14 consumed MFD. These data, along with recovery data and compared to information available for other probiotics belonging to the same species [52], indicated CRL1505's ability to survive passage through the GIT in adults even without any protective effect of foods or microencapsulation.

These data are not surprising because matrices can offer probiotics protection throughout the GIT [56], although the effect of the matrix can be strain dependent.

Analysis of the taxonomic profile of fecal samples from the study did not reveal significant differences in α‐diversity indices or β‐diversity between the treatment groups. These findings align with existing literature, as the participants enrolled in the study were healthy individuals, and a stable and resilient gut microbiota is characteristic of a healthy population [57, 58, 59, 60]. However, unlike the 16S profiling approach, a shotgun metagenomic analysis could have revealed strain‐level variations with possible implications on the metabolic potential of the gut microbiota. Moreover, analysis of the fecal microbiota of volunteers considering pre‐ and post‐consumption of FDC, OFD, or MFD allowed us to detect an increase of the species *L. rhamnosus* only in OFD and MFD. In contrast, a nonsignificant increase of this species was measured after FDC consumption, unlike what was shown by quantifying *L. rhamnosus* CRL1505 by qPCR. These apparently varying results could be explained by the lower sensitivity of 16S profiling compared to a qPCR approach targeting a specific strain in a complex community [61]. However, a 16S rRNA‐based microbial profiling lacks functional information [62]. Therefore, it remains to be investigated whether any metabolic changes (e.g., short‐chain fatty acid content) were introduced by the three different products to the microbiome of the individuals.

## 5 Conclusions

In conclusion, this work evaluated for the first time the ability of *L. rhamnosus* CRL1505 strain to survive after human GIT. The double crossover study comparing FDC versus OFD and FDC versus MFD allowed us to demonstrate the positive effect of food matrix, namely milk and oats, on the survival of probiotic cells. However, our data confirmed the strong ability of the CRL1505 strain to survive after the GIT both as FDC (without any food matrix protection) and as a probiotic‐enriched fermented beverage. Due to probiotic recovery after a 2‐week washout in some of the volunteers, further studies could be focused on the persistence ability of CRL1505 by increasing the monitoring of the follow‐up phase. This is the first comprehensive analysis of the survival of *L. rhamnosus* CRL1505 through the GIT in an interventional study, elucidating the recovery of this probiotic strain in the fecal samples of healthy volunteers.

## Conflicts of Interest

The authors declare no conflicts of interest.

## Supporting information




**Supporting file 1**: mnfr70272‐sup‐0001‐SuppMat.docx.

## Data Availability

The data that support the findings of this study are openly available in European Nucleotide Archive at https://www.ebi.ac.uk/ena/browser/view/PRJEB89811, reference number PRJEB89811.

## References

[1] Food and Agricultural Organization of the United Nations and World Health Organization , Joint FAO/WHO Working Group Report on Drafting Guidelines for the Evaluation of Probiotics in Food (Food and Agricultural Organization of the United Nations, 2001).

[2] C. Hill , F. Guarner , G. Reid , et al., “The International Scientific Association for Probiotics and Prebiotics Consensus Statement on the Scope and Appropriate Use of the Term Probiotic,” Nature Reviews Gastroenterology & Hepatology 11 (2014): 506–514. 24912386 10.1038/nrgastro.2014.66

[3] M. Kechagia , D. Basoulis , S. Konstantopoulou , et al., “Health Benefits of Probiotics: A Review,” ISRN Nutrition 2013 (2013): 481651. 24959545 10.5402/2013/481651 PMC4045285

[4] K. Fenster , B. Freeburg , C. Hollard , C. Wong , R. Rønhave Laursen , and A. C. Ouwehand , “The Production and Delivery of Probiotics: A Review of a Practical Approach,” Microorganisms 7 (2019): 83. 30884906 10.3390/microorganisms7030083 PMC6463069

[5] R. D. C. S. Ranadheera , S. K. Baines , and M. C. Adams , “Importance of food in probiotic efficacy,” Food Research International 43 (2010): 1–7.

[6] V. Valli , A. Taccari , M. Di Nunzio , F. Danesi , and A. Bordoni , P. J. Espitia , R. A. Batista , H. M. Azeredo , and C. G. Otoni , “Probiotics and their potential in probiotic efficacy,” Food Research International 90 (2016): 42–52. 29195890 10.1016/j.foodres.2016.10.026

[7] P. Kandylis , K. Pissaridi , A. Bekatorou , M. Kanellaki , and A. A. Koutinas , “Dairy and Non‐Dairy Probiotic Beverages,” Current Opinion in Food Science 7 (2016): 58–63.

[8] C. S. Ranadheera , J. K. Vidanarachchi , R. S. Rocha , A. G. Cruz , and S. Ajlouni , “Probiotic Delivery Through Fermentation: Dairy vs. Non‐Dairy Beverages,” Fermentation 3 (2017): 67.

[9] B. Vijaya Kumar , S. V. N. Vijayendra , and O. V. S. Reddy , “Trends in dairy and non‐dairy probiotic products ‐ a review,” Journal of Food Science and Technology 52 (2015): 6112. 26396359 10.1007/s13197-015-1795-2 PMC4573104

[10] A. C. Domínguez‐Murillo and J. E. Urías‐Silvas , “Plant‐Based Milk Substitutes as Probiotic Vehicles: Health Effect and Survival, a Review,” Food Chemistry Advances 5 (2024): 100830.

[11] S. Sethi , S. K. Tyagi , and R. K. Anurag , “Plant‐based milk alternatives an emerging segment of functional beverages: a review,” Journal of Food Science and Technology 53 (2016): 3408–3423. 27777447 10.1007/s13197-016-2328-3 PMC5069255

[12] N. Sharma , N. Yeasmen , L. Dube , and V. Orsat , “Rise of Plant‐Based Beverages: A Consumer‐Driven Perspective,” Food Research International 40 (2024): 3315–3341.

[13] K. D. Kaur , A. Jha , L. Sabikhi , and A. K. Singh , “Significance of Coarse Cereals in Health and Nutrition: A Review,” Journal of Food Science and Technology 51 (2014): 1429–1441. 25114333 10.1007/s13197-011-0612-9 PMC4108649

[14] M. Gupta and B. K. Bajaj , “Development of Fermented Oat Flour Beverage as a Potential Probiotic Vehicle,” Food Bioscience 20 (2017): 104–109.

[15] D. Paudel , B. Dhungana , M. Caffe , and P. Krishnan , “A Review of Health‐Beneficial Properties of Oats,” Foods 10 (2021): 2591. 34828872 10.3390/foods10112591 PMC8625765

[16] S. Salva , J. Villena , and S. Alvarez , “Immunomodulatory Activity of *Lactobacillus rhamnosus* Strains Isolated From Goat Milk: Impact on Intestinal and respiratory Infections,” International Journal of Food Microbiology 141 (2010): 82–89. 20395002 10.1016/j.ijfoodmicro.2010.03.013

[17] M. P. Taranto , J. Villena , S. Salva , et al., “Draft Genome Sequence of *Lactobacillus rhamnosus* CRL1505, an Immunobiotic Strain Used in Social Food Programs in Argentina,” *Genome Announcements* 1 (2013): e00627–13. 10.1128/genomeA.00627-13 PMC3744685 23950129

[18] S. D. Maidana , Y. Imamura , M. Elean , et al., “Oral Administration of *Lacticaseibacillus rhamnosus* CRL1505 Modulates Lung Innate Immune Response Against *Klebsiella pneumoniae* ST25,” Microorganisms 11 (2023): 1148. 37317122 10.3390/microorganisms11051148 PMC10222716

[19] F. R. Tonetti , M. A. Islam , M. G. Vizoso‐Pinto , H. Takahashi , H. Kitazawa , and J. Villena , “Nasal Priming With Immunobiotic Lactobacilli Improves the Adaptive Immune Response Against Influenza Virus,” International Immunopharmacology 78 (2020): 106115. 31841753 10.1016/j.intimp.2019.106115

[20] J. Villena and H. Kitazawa , “The Modulation of Mucosal Antiviral Immunity by Immunobiotics: Could They Offer Any Benefit in the SARS‐CoV‐2 Pandemic?” Frontiers in Physiology 11 (2020): 699. 32670091 10.3389/fphys.2020.00699 PMC7326040

[21] H. Zelaya , A. Tada , M. G. Vizoso‐Pinto , et al., “Nasal Priming With Immunobiotic *Lactobacillus rhamnosus* Modulates Inflammation–Coagulation Interactions and Reduces Influenza Virus‐Associated Pulmonary Damage,” Inflammation Research 64 (2015):] 589–602. 26072063 10.1007/s00011-015-0837-6

[22] J. Villena , S. Salva , M. Nuñez , et al., “Probiotics for everyone! The novel immunobiotic Lactobacillus rhamnosus CRL 1505 and the beginning of Social Programs in Argentina,” International Journal of Biotechnology for Wellness Industries, 1 (2012) 000.

[23] P. Aureli , A. Fiore , C. Scalfaro , and G. Franciosa , “Microbiological and molecular methods for analysis of probiotic based food supplements for human consumption,” Rapporti ISTISAN 8 (2008): 63.

[24] V. Taverniti , R. Koirala , A. Dalla Via , et al., “Effect of Cell Concentration on the Persistence in the Human Intestine of Four Probiotic Strains Administered Through a Multispecies Formulation,” Nutrients 11 (2019): 285. 30699901 10.3390/nu11020285 PMC6412360

[25] A. Martinović , M. Chittaro , D. Mora , and S. Arioli , “The Ability of *Streptococcus thermophilus* BT01 to Modulate Urease Activity in Healthy Subjects′ Fecal Samples Depends on the Biomass Production Process,” Molecular Nutrition & Food Research 67 (2023): 2200529. 10.1002/mnfr.202200529 36708131

[26] L. J. H. Ward and M. J. Timmins , “Differentiation of Lactobacillus Casei, *Lactobacillus paracasei* and *Lactobacillus rhamnosus* by Polymerase Chain Reaction,” Letters in Applied Microbiology 29 (1999): 90–92. 10499296 10.1046/j.1365-2672.1999.00586.x

[27] M. A. Elovitz , P. Gajer , V. Riis , et al., “Cervicovaginal Microbiota and Local Immune Response Modulate the Risk of Spontaneous Preterm Delivery,” Nature Communications 10 (2019): 1305. 10.1038/s41467-019-09285-9 PMC6428888 30899005

[28] E. Bolyen , J. R. Rideout , M. R. Dillon , et al., “Reproducible, Interactive, Scalable and Extensible Microbiome Data Science Using QIIME 2,” Nature Biotechnology 37 (2019): 852–857. 10.1038/s41587-019-0209-9 PMC7015180 31341288

[29] B. J. Callahan , P. J. McMurdie , M. J. Rosen , A. W. Han , A. J. A. Johnson , and S. P. Holmes , “DADA2: High‐Resolution Sample Inference From Illumina Amplicon Data,” Nature Methods 13 (2016): 581–583. 27214047 10.1038/nmeth.3869 PMC4927377

[30] N. Segata , J. Izard , L. Waldron , et al., “Metagenomic Biomarker Discovery and Explanation,” Genome Biology 12 (2011): R60. 21702898 10.1186/gb-2011-12-6-r60 PMC3218848

[31] Z. Ba , Y. Lee , H. Meng , et al., “Matrix Effects on the Delivery Efficacy of *Bifidobacterium animalis* subsp. Lactis BB‐12 on Fecal Microbiota, Gut Transit Time, and Short‐Chain Fatty Acids in Healthy Young Adults,” mSphere 6 (2021): 0008421. 10.1128/mSphere.00084-21 PMC8386398 34232082

[32] M. R. Damián , N. G. Cortes‐Perez , E. T. Quintana , et al., “Functional Foods, Nutraceuticals and Probiotics: A Focus on Human Health,” Microorganisms 10 (2022): 1065. 35630507 10.3390/microorganisms10051065 PMC9143759

[33] P. Matouskova , J. Hoova , P. Rysavka , and I. Marova , “Stress Effect of Food Matrices on Viability of Probiotic Cells During Model Digestion,” Microorganisms 9 (2021): 1625. 34442704 10.3390/microorganisms9081625 PMC8401621

[34] N. Yeung , S. D. Forssten , M. T. Saarinen , M. Anjum , and A. C. Ouwehand , “The Effect of Delivery Matrix on *Bifidobacterium animalis* subsp. Lactis HN019 Survival Through In Vitro Human Digestion,” Nutrients 15 (2023): 3541. 37630731 10.3390/nu15163541 PMC10459543

[35] S. J. Lahtinen , “Probiotic viability ‐ does it matter?,” Microbial Ecology in Health and Disease 23 (2012): 18567. 10.3402/mehd.v23i0.18567 PMC3747757 23990833

[36] S. Arioli , R. Koirala , V. Taverniti , W. Fiore , and S. Guglielmetti , “Quantitative Recovery of Viable *Lactobacillus paracasei* CNCM I‐1572 (*L. casei* DG®) After Gastrointestinal Passage in Healthy Adults,” Frontiers in Microbiology 9 (2018): 1720. 30116228 10.3389/fmicb.2018.01720 PMC6083036

[37] T. Ahlroos and S. Tynkkynen , “Quantitative Strain‐Specific Detection of *Lactobacillus rhamnosus* GG in Human Faecal Samples by Real‐Time PCR,” Journal of Applied Microbiology 106 (2009): 506–514. 19200317 10.1111/j.1365-2672.2008.04018.x

[38] R. Wang , S. Chen , J. Jin , et al., “Survival of Lactobacillus casei strain Shirota in the intestines of healthy Chinese adults,” Microbiology and Immunology 59 (2015): 268–276. 25707300 10.1111/1348-0421.12249

[39] R. Oozeer , A. Leplingard , D. D. Mater , et al., “Survival of *Lactobacillus casei* in the Human Digestive Tract After Consumption of Fermented Milk,” Applied and Environmental Microbiology 72 (2006): 5615–5617. 16885316 10.1128/AEM.00722-06 PMC1538725

[40] C. L. Gerez , G. Font de Valdez , M. L. Gigante , and C. R. F. Grosso , “Whey Protein Coating Bead Improves the Survival of the Probiotic *Lactobacillus rhamnosus* CRL 1505 to Low pH,” Letters in Applied Microbiology 54 (2012): 552–556. 22448978 10.1111/j.1472-765X.2012.03247.x

[41] G. Reid , R. Kort , S. Alvarez , et al., “Expanding the Reach of Probiotics Through Social Enterprises,” Beneficial Microbes 9 (2018): 707–715. 29798708 10.3920/BM2018.0015

[42] M. A. Correa Deza , S. Salva , M. Grillo‐Puertas , G. M. Font , and C. L. Gerez , “Effect of Culture Parameters on the Heat Tolerance and Inorganic Polyphosphate Accumulation by *Lacticaseibacillus rhamnosus* CRL1505, a Multifunctional Bacterium,” World Journal of Microbiology and Biotechnology 39 (2023): 182. 37145244 10.1007/s11274-023-03625-0 PMC10159826

[43] H. Zelaya , K. Tsukida , E. Chiba , et al., “Immunobiotic *Lactobacilli* Reduce Viral‐Associated Pulmonary Damage Through the Modulation of Inflammation–Coagulation Interactions,” International Immunopharmacology 19 (2014): 161–173. 24394565 10.1016/j.intimp.2013.12.020

[44] M. I. Chávez de la Vega , S. Alatorre‐Santamaría , L. Gómez‐Ruiz , et al., “Influence of Oat β‐Glucan on the Survival and Proteolytic Activity of *Lactobacillus rhamnosus* GG in Milk Fermentation: Optimization by Response Surface,” Fermentation 7 (2021): 210.

[45] I. M. Sims , J. L. Ryan , and S. H. Kim , “In Vitro Fermentation of Prebiotic Oligosaccharides by *Bifidobacterium lactis* HN019 and *Lactobacillus* spp,” Anaerobe 25 (2014): 11–17. 24239979 10.1016/j.anaerobe.2013.11.001

[46] M. Saarela , I. Virkajärvi , L. Nohynek , A. Vaari , and J. Mättö , “Fibres as Carriers for *Lactobacillus rhamnosus* During Freeze‐Drying and Storage in Apple Juice and Chocolate‐Coated Breakfast Cereals,” International Journal of Food Microbiology 112 (2006): 171–178. 16844253 10.1016/j.ijfoodmicro.2006.05.019

[47] C. Barthow , F. Hood , E. McKinlay , et al., “Food 4 Health—He Oranga Kai: Assessing the Efficacy, Acceptability and Economic Implications of *Lactobacillus rhamnosus* HN001 and β‐Glucan to Improve Glycated Haemoglobin, Metabolic Health, and General Well‐Being in Adults With Pre‐Diabetes: Study Protocol for a 2 × 2 Factorial Design, Parallel Group, Placebo‐Controlled Randomized Controlled Trial, With Embedded Qualitative Study and Economic Analysis,” Trials 20 (2019): 464. 31358022 10.1186/s13063-019-3553-7 PMC6664750

[48] D. Kumar , M. K. Lal , S. Dutt , et al., “Functional Fermented Probiotics, Prebiotics, and Synbiotics From Non‐Dairy Products: A Perspective From Nutraceutical,” Molecular Nutrition & Food Research 66 (2022): 2101059. 10.1002/mnfr.202101059 35616160

[49] M. Céspedes , P. Cárdenas , M. Staffolani , M. C. Ciappini , and G. Vinderola , “Performance in nondairy drinks of probiotics L. casei strains usually employed in dairy products,” Journal of Food Science 78 (2013): M75662. 10.1111/1750-3841.12092 23527588

[50] M. Saxelin , S. Tynkkynen , T. Mattila‐Sandholm , and W. M. de Vos , “Probiotic and Other Functional Microbes: From Markets to Mechanisms,” Current Opinion in Biotechnology 16 (2005): 204–211. 15831388 10.1016/j.copbio.2005.02.003

[51] M. Ziarno and D. Zaręba , “Effects of milk components and food additives on survival of three bifidobacteria strains in fermented milk under simulated gastrointestinal tract conditions,” Microbial Ecology in Health and Disease 26 (2015): 27812. 26546945 10.3402/mehd.v26.27812 PMC4636862

[52] L. Morelli and P. Pellegrino , “A Critical Evaluation of the Factors Affecting the Survival and Persistence of Beneficial Bacteria in Healthy Adults,” Beneficial Microbes 12 (2021): 15–25. 34323162 10.3920/BM2021.0017

[53] R. Kumar and A. Kumar , “Influence of inulin and maltodextrin on survival of probiotic bacteria during spray drying,” Journal of Food Science and Technology 52 (2015): 4081–4086.

[54] G. Vinderola , M. F. Zacarías , W. Bockelmann , H. Neve , J. Reinheimer , and K. J. Heller , “Preservation of Functionality of *Bifidobacterium animalis* subsp. Lactis INL1 After Incorporation of Freeze‐Dried Cells Into Different Food Matrices,” Food Microbiology 30 (2012): 274–280. 22265312 10.1016/j.fm.2011.12.004

[55] M. H. Saarela , H. L. Alakomi , A. Puhakka , and J. Mättö , “Effect of the Fermentation pH on the Storage Stability of *Lactobacillus rhamnosus* Preparations and Suitability of In Vitro Analyses of Cell Physiological Functions to Predict It,” Journal of Applied Microbiology 106 (2009): 1204–1212. 19191949 10.1111/j.1365-2672.2008.04089.x

[56] J. Flach , M. B. van der Waal , M. van den Nieuwboer , E. Claassen , and O. F. Larsen , “The Underexposed Role of Food Matrices in Probiotic Products: Reviewing the Relationship Between Carrier Matrices and Product Parameters,” Critical Reviews in Food Science and Nutrition 58 (2018): 2570–2584. 28609116 10.1080/10408398.2017.1334624

[57] K. Z. Coyte , J. Schluter , and K. R. Foster , “The Ecology of the Microbiome: Networks, Competition, and Stability,” Science 350 (2015): 663–666. 26542567 10.1126/science.aad2602

[58] C. A. Lozupone , J. I. Stombaugh , J. I. Gordon , J. K. Jansson , and R. Knight , “Diversity, Stability and Resilience of the Human Gut Microbiota,” Nature 489 (2012): 220–230. 22972295 10.1038/nature11550 PMC3577372

[59] F. Sommer , J. M. Anderson , R. Bharti , J. Raes , and P. Rosenstiel , “The Resilience of the Intestinal Microbiota Influences Health and Disease,” Nature Reviews Microbiology 15 (2017): 630–638. 28626231 10.1038/nrmicro.2017.58

[60] “The Human Microbiome Project Consortium, Structure, function and diversity of the healthy human microbiome,” Nature 486 (2012): 207–214. 22699609 10.1038/nature11234 PMC3564958

[61] C. Ferreira , S. Otani , F. M. Aarestrup , and C. M. Manaia , “Quantitative PCR Versus Metagenomics for Monitoring Antibiotic Resistance Genes: Balancing High Sensitivity and Broad Coverage,” FEMS Microbes 4 (2023): xtad008. 37333442 10.1093/femsmc/xtad008 PMC10117749

[62] M. S. Matchado , M. Ruhlemann , S. Reitmeier , et al., “On the Limits of 16S rRNA Gene‐Based Metagenome Prediction and Functional Profiling,” Microbial Genomics 10 (2024): 001203. 38421266 10.1099/mgen.0.001203 PMC10926695

