# Supplementary material for: Effect of the Food Matrix on the Survival to the Gastrointestinal Transit of Lacticaseibacillus rhamnosus CRL1505: A Randomized, Controlled, Crossover Study
Source: Mol Nutr Food Res. 2025 Sep 20;69(23):e70272. doi: 10.1002/mnfr.70272 (PMC12666742; doi:10.1002/mnfr.70272)
Supplement: Supplementary file 1 — Supporting file 1: mnfr70272‐sup‐0001‐SuppMat.docx. [file MNFR-69-e70272-s001.docx]

**Effect of the food matrix on the survival to the gastrointestinal transit of *Lacticaseibacillus rhamnosus* CRL1505: a randomized, controlled, crossover study**

Nicola Mangieri^1*^, Viola Termine^1*^, Giorgio Gargari^1^, Nicolò Fornasari^1^, Michele Isotti^1^, Susana Salva^3^, Julio Villena^3^, María Pía Taranto^3^, Valentina Taverniti^2^, Susana Álvarez^3^, Graciela Font^3^, Ylenia Zanchetta^1^, Stefania Arioli^1#^, Diego Mora^1^

^1^Department of Food, Environmental and Nutritional Sciences, University of Milan, via G. Celoria 2, 20133 Milan Italy

^2^Sacco Srl, via A. Manzoni 29/A, 22071 Cadorago, Como, Italy

^3^Centro de Referencia para Lactobacilos (CERELA-CONICET), Chacabuco 145, San Miguel de Tucumán, T4000ILC Tucumán, Argentina

*These authors contributed equally to this study

^#^corresponding author:

Stefania Arioli

Department of Food, Environmental and Nutritional Sciences

University of Milan

Via Giovanni Celoria 2, 20133, Milan Italy

[stefania.arioli@unimi.it](mailto:stefania.arioli@unimi.it)

**Supplemental material**

**Table S1.** Set up of the medium for the cultivable recovery of CRL1505 cells in fecal samples of the *survivor* study. CRL1505 pure culture was cultured on MRS 0, 1, and 10 µg ml^-1^ vancomycin. Plates were incubated at 37 °C for 72 h, in anaerobic conditions, the values are the mean of three technical replicates, ±: standard deviation.

|  | **Medium** | | |
| --- | --- | --- | --- |
|  | **MRS 0v** | **MRS 1v** | **MRS 10v** |
| Average CRL1505 CFU g^-1^ ± std. dev | 4.23 x 10^11^ ± 0.52 | 4.57 x 10^11^ ± 0.99 | 3.67 x 10^11^ ± 0.62 |

*no significative differences have been detected according to one-way ANOVA (p. value = 0.097)*

|  | **Temperature of incubation** | |
| --- | --- | --- |
|  | **37 °C** | **43 °C** |
| Average CRL1505 CFU g^-1^ ± std. dev | 3.84 x10^11^ ± 0.67 | 3.53 x 10^11^ ± 0.57 |

*No significative differences have been detected according to a paired t-Test (p. value = 0.33)*

**Table S2**. Viable quantification of *L. delbrueckii* subsp. *bulgaricus* Ldb1 and *S. thermophilus* St1 in OFD and MFD evaluated at the 1^st^ and 7^th^ day of consumption. For this analysis different bottles of each batch were randomly selected; the values are the mean of three biological replicates ±: standard deviation.

| ***Lactobacillus delbrueckii subsp. bulgaricus* Ldb1** | | | | | |
| --- | --- | --- | --- | --- | --- |
| **Food matrix** | **Batch** | **log_10_ CFU/50 mL**  **1st day** | **log_10_ CFU/50 mL**  **7th day** | **Average**  **log_10_ CFU/50 ml** | |
| MFD | 1st | 10.50 ± 0.06 | 10.20 ± 0.00 | | 10.35 ± 0.21 |
| MFD | 2nd | 9.38 ± 0.04 | 9.68 ± 0.013 | | 9.53 ± 0.21 |
| **MFD** | **Average** | **9.94 ± 0.61** | **9.94 ± 0.28** | | **9.94 ± 0.45** |

*No significant differences have been detected in the CFU comparing the 1° day and the 7° day of consumption of MDF, according to a paired t-Test (p.value = 0.37)*

| ***Streptococcus thermophilus* St1** | | | | |
| --- | --- | --- | --- | --- |
| **Food matrix** | **Batch** | **log_10_ CFU/50 mL**  **1st day** | **log_10_ CFU/50 mL**  **7th day** | **Average**  **log_10_ CFU/50 ml** |
| OFD | 1° | 9.86 ± 0.03 | 9.81 ± 0.01 | 9.84 ± 0.04 |
| OFD | 2° | 9.57 ± 0.03 | 10.02± 0.01 | 9.87 ± 0.42 |
| **OFD** | **Average** | **9.72 ± 0.16** | **10.00 ± 0.20*** | **9.85 ± 0.22** |
| MFD | 1° | 10.02 ± 0.02 | 10.60 ± 0.08 | 10.31 ± 0.33 |
| MFD | 2° | 10.02 ± 0.01 | 9.84 ± 0.02 | 9.55 ± 0.11 |
| **MFD** | **Average** | **10.02 ± 0.01** | **10.30 ± 0.42** | **10.13 ± 0.25** |

*A significant difference (p.value = 0.03) has been detected in the CFU at 1st and the 7^th^ day of consumption of OFD, according to a paired t-Test. No significant differences have been detected in the CFU at the 1^st^ and 7^th^ day of consumption of MFD, according to a paired t-Test (p.value = 0.06).*

**Table S3**. Basic characteristics of study’s volunteers.

| **Subject** | **Sex** | **Age** |
| --- | --- | --- |
| **(n = 20)** | **(10F/10M)** | **(18-60 years)** |
| **A** | F | 48 |
| **B** | M | 53 |
| **C** | F | 53 |
| **D** | F | 57 |
| **E** | F | 51 |
| **F** | M | 27 |
| **G** | M | 43 |
| **H** | M | 32 |
| **I** | M | 32 |
| **L** | F | 27 |
| **N** | M | 26 |
| **O** | M | 23 |
| **P** | F | 22 |
| **R** | F | 28 |
| **S** | F | 30 |
| **T** | M | 24 |
| **U** | M | 26 |
| **V** | F | 30 |
| **X** | F | 25 |
| **Z** | M | 34 |
| **Mean age ± std.dev.** |  | 35 ± 12 |

**Table S6**. Defecation frequency based on Bristol stool scale (Wilcoxon test). n= total number of evacuations reported during the week of probiotic consumption.

| **Wilcoxon test on one week's results** | **p-value** | **Median** | |
| --- | --- | --- | --- |
| **MFD vs OFD n=266** |  | **MFD** | **OFD** |
| **Number of evacuations** | **0.31** | **1** | **1** |
| **Type of feces** | **0.17** | **3** | **4** |
|  |  |  |  |
| **OFD vs FDC n=259** |  | **OFD** | **FDC** |
| **Number of evacuations** | **0.27** | **1** | **1** |
| **Type of feces** | **0.93** | **4** | **4** |
|  |  |  |  |
| **MFD vs FDC n=266** |  | **MFD** | **FDC** |
| **Number of evacuations** | **0.57** | **1** | **1** |
| **Type of feces** | **0.85** | **3** | **3** |
|  |  |  |  |
| **FDC vs FDC n=259** |  | **FDC** | **FDC** |
| **Number of evacuations** | **0.85** | **1** | **1** |
| **Type of feces** | **0.12** | **4** | **3** |

**Table S7**. Stool consistency based on delta number of evacuation between the begin and the end of each probiotic treatment (Wilcoxon test)

|  | **Wilcoxon** | **Numbers of** | **Average** | | **Median** | |
| --- | --- | --- | --- | --- | --- | --- |
| **MFD vs OFD** | **p.values** | **observation** | **MFD** | **OFD** | **MFD** | **OFD** |
| **Number of evacuations** | 0.819 | 36.000 | 0.000 | 0.111 | 0.000 | 0.000 |
| **Type of feces** | 0.162 | 36.000 | 0.222 | 0.722 | 0.000 | 0.500 |
| **MFD vs FDC** | **p.values** | **observation** | **MFD** | **FDC** | **MFD** | **FDC** |
| **Number of evacuations** | 0.487 | 36.000 | 0.000 | -0.278 | 0.000 | -0.500 |
| **Type of feces** | 0.217 | 36.000 | 0.222 | -0.278 | 0.000 | 0.000 |
| **MFD vs FDC** | **p.values** | **observation** | **MFD** | **FDC** | **MFD** | **FDC** |
| **Number of evacuations** | 0.626 | 36.000 | 0.000 | 0.167 | 0.000 | 0.000 |
| **Type of feces** | 0.821 | 36.000 | 0.222 | 0.278 | 0.000 | 0.000 |
| **OFD vs FDC** | **p.values** | **observation** | **OFD** | **FDC** | **OFD** | **FDC** |
| **Number of evacuations** | 0.236236 | 36 | 0.111111 | -0.27778 | 0 | -0.5 |
| **Type of feces** | 0.069601 | 36 | 0.722222 | -0.27778 | 0.5 | 0 |
| **OFD vs FDC** | **p.values** | **observation** | **OFD** | **FDC** | **OFD** | **FDC** |
| **Number of evacuations** | 0.857 | 36.000 | 0.111 | 0.167 | 0.000 | 0.000 |
| **Type of feces** | 0.358 | 36.000 | 0.722 | 0.278 | 0.500 | 0.000 |
| **FDC vs FDC** | **p.values** | **observation** | **FDC** | **FDC** | **FDC** | **FDC** |
| **Number of evacuations** | 0.237933 | 36.000 | -0.27778 | 0.166667 | -0.5 | 0 |
| **Type of feces** | 0.285508 | 36.000 | -0.27778 | 0.277778 | 0 | 0 |

**Table S6**: Absolute quantification of CRL1505 before consumption and at the end of consumption of each product (Wilcoxon test)

| **Wilcoxon test** | **p-value** | **Median** | |
| --- | --- | --- | --- |
| **MFD n=38** |  | **V8+V11** | **V10+V13** |
| **log_10_ cells/g** | 5.34E-05 | 0 | 7.13 |
|  |  |  |  |
| **OFD n=36** |  | **V1+V4** | **V3+V6** |
| **log_10_ cells/g** | 0.00032 | 0 | 6.70 |
|  |  |  |  |
| **FDC n=70** |  | **V1+V4**  **V8+V11** | **V3+V6**  **V10+V13** |
| **log_10_ cells/g** | 4.89E-05 | 0 | 4.80 |

**Figure S1**. Standard curve for total CRL1505 cell quantification in fecal samples Cq values were plotted versus log_10_ of the number of cells added to aliquots of the same fecal sample. Limit of quantification (LOQ) and R^2^ are indicated.
